# The Ste20 Homologue FvM4K1 Regulates Organ Size via Hippo Signalling Pathway in Woodland Strawberry ( *Fragaria vesca* )

**DOI:** 10.1111/pbi.70286

**Published:** 2025-08-04

**Authors:** Si Gu, Xinghua Nie, Ling Qin, Yu Xing, Baoxiu Qi

**Affiliations:** ^1^ School of Pharmacy and Biomolecular Science Liverpool John Moores University Liverpool UK; ^2^ College of Plant Science and Technology, Beijing Advanced Innovation Center for Tree Breeding by Molecular Design, Beijing Key Laboratory for Agricultural Application and New Technique Beijing University of Agriculture Beijing China

**Keywords:** *Fragaria vesca*, Hippo signalling pathway, MAP4K, MOB1, organ size, phosphorylation, Ste20

## Abstract

Strawberry fruit size is critical for its marketability. However, organ size control is a complex process regulated by various signalling pathways. In animals, the Hippo signalling pathway acts as a negative regulator of organ size, with Ste20 kinase being a key component. Mutation in Ste20 causes excessive cell proliferation and enlarged organs. In this study, FvM4K1, a Ste20‐like kinase from woodland strawberry ( *Fragaria vesca* ), is identified. FvM4K1 partially restores defects in a yeast Ste20 mutant *ste20Δ* and fully rescues growth in Arabidopsis mutant *atsik1‐4* lacking its Ste20 homologue, AtSIK1, underscoring its functional conservation. Downregulation of FvM4K1 by RNAi in woodland strawberry leads to smaller plants and fruits resulting from reduced cell size and number, while overexpression increases organ size, indicating a positive role in organ size control which contrasts with the negative role of Ste20 in other organisms. FvM4K1 autophosphorylates, with Lys269 and Thr396 being critical for its function. FvM4K1 interacts with FvMOB1A and FvMOB1B, components of the Hippo signalling pathway, and phosphorylates them at Thr35 and Thr36, respectively. These findings provide novel insights into the mechanisms underlying fruit and organ size control in woodland strawberry and contribute to our understanding of the Hippo signalling pathway in higher plants, a pathway that remains largely unexplored. It also opens new avenues for exploring the regulatory function of the Hippo pathway in plant development and potentially informs biotechnological strategies for crop improvement.

## 1 Introduction

Strawberry is a valuable crop and its fruit size is a key factor in determining its market value. Organ size control is a complex and highly coordinated process influenced by various signalling pathways. The Hippo pathway, initially discovered in fruit fly ( *Drosophila melanogaster* ), is a critical regulator of organ size in animals (Wu et al. 2003). Mutations in the Hippo signalling pathway can lead to uncontrolled cell proliferation and cancer (Hwang et al. 2019). However, its function in plants remains largely unknown.

Study in yeast ( *Saccharomyces cerevisiae* ) has shown that the serine/threonine kinase family Ste20 (Sterile 20) is a central component of the Hippo signalling pathway. Ste20 includes two subfamilies, the p21 activated kinases (PAKs) and the germinal centre kinases (GCKs), which differ in the location of their kinase domains (KDs): PAKs have a C‐terminal, while GCKs have an N‐terminal KD (Boyce and Andrianopoulos 2011). The yeast Ste20p is a typical member of the PAK subfamily and plays important roles including mating regulation (Wu et al. 1995), bud site selection (Sheu et al. 2000) and mitosis exit (Höfken and Schiebel 2002) whilst Cdc15p of the GCKs is one of the core components in the mitotic exit network (Rock et al. 2013). Hippo (Hpo), a FCK and homologue of Cdc15p in flies, is involved in controlling organ size via proliferation and apoptosis (Wu et al. 2003). Flies have five core components in the Hippo signalling pathway: Hpo, its scaffolding protein Salvador (Sav), the Nuclear Dbf2‐related (NDR) kinase Warts (Wts), its helper Mps One Binder (MOB1) protein Mats, and the transcription factor Yorkie (Yki). Hpo, in association with Sav, phosphorylates the downstream Wts/Mats complex. This is followed by phosphorylation and inactivation of the transcription factor Yki in the cytoplasm by 14‐3‐3 mediated ubiquitin degradation, resulting in growth inhibition (Zhao et al. 2011; Zheng and Pan 2019). When Hippo signalling is inactivated, Yki remains unphosphorylated and translocates to the nucleus where it binds with Scalloped (sd) to activate the expression of target genes, including *cyclin‐E* (*cycE*), *diap1* and *bantam*. This activation promotes proliferation and inhibits apoptosis, resulting in larger wings and other organs due to excessive cell growth and proliferation (Zhao et al. 2011; Zheng and Pan 2019). In humans, the Ste20 kinase Mst1/2 interacts with and phosphorylates the scaffold protein WW45, leading to phosphorylation and activation of the downstream complex consisting of Lats1/2 and MOB1s. The transcription factor Yes‐associated protein (YAP) and the transcriptional co‐activator TAZ (PDZ‐binding motif) are inactivated by phosphorylation by the LATS1/2 and MOB1 complex, promoting cell proliferation and cancer (Zheng and Pan 2019). Recent advances in the understanding of the Hippo signalling pathway have led to the identification of more than 30 components (Zhao et al. 2011; Zheng and Pan 2019).

Studies on the Hippo signalling pathway in plants are limited and primarily focused on the model plant Arabidopsis. Only a few components have been identified so far, including the Ste20 kinase AtSIK1 (Xiong et al. 2016), the MOB1 homologues AtMOB1A and AtMOB1B (Guo et al. 2020) and eight NDR homologues (Zhou et al. 2021). Mutants lacking *AtSIK1* exhibited inhibited growth characterised by reduced plant height, smaller leaves and flowers, due to reduced cell size and number. This suggested that AtSIK1 plays a role in controlling organ size by regulating cell expansion and proliferation (Xiong et al. 2016). AtSIK1 interacts with AtMOB1A&B, homologues of MOB1 (Xiong et al. 2016). The double mutant *mob1a−/−1b+/−* displayed a similar phenotype to *sik1−/−*, while the double mutant *sik1−/−mob1a+/−* was much smaller than the respective single mutants. In addition, the homozygous double mutant *sik1−/−mob1a−/−* struggled to survive (Guo et al. 2020). These findings suggest that AtSIK1 and AtMOB1s function in the same pathway and positively regulate organ size via the Hippo signalling pathway, different to the negative role of the Hippo signalling pathway in animals.

AtNDRs are primarily required for pollen development, fertilisation and seed set in Arabidopsis (Yoon et al. 2021). There are eight AtNDRs that appear to function redundantly, as single and double mutants of *atndr2*, *atndr4* and *atndr5* show no noticeable phenotype. However, the *atndr2/atndr4/atndr5* triple mutant produces much shorter siliques with reduced fertility than wild type (WT; Zhou et al. 2021). NDR2/4/5 interact with MOB1 proteins and regulate pollen development (Zhou et al. 2021). However, the interaction between AtSIK1 and AtNDRs has not yet been explored, although the interaction between the homologous proteins Ste20 and NDRs in yeast and animals is verified (Rock et al. 2013; Zhao et al. 2011; Zheng and Pan 2019).

Therefore, the current understanding of the Hippo signalling pathway in Arabidopsis suggests that it may function differently from its role in yeast, flies and animals. While Hippo signalling in other organisms typically acts to inhibit cell proliferation and limit organ size, in Arabidopsis it appears to positively regulate cell proliferation and growth. Additionally, far fewer components in this pathway have been identified in Arabidopsis than in other species, and importantly, it remains unclear how these limited components coordinate cell proliferation and organ size. Validating the presence and function of homologue proteins in the Hippo signalling pathway in another plant, especially in a crop species, could provide valuable insights into their role in organ size regulation, a key trait in plant breeding.

The aim of this study is to investigate the role of the Ste20 kinase homologue FvM4K1 in woodland strawberry, a model plant for the Rosaceae family. Through complementation studies in both yeast and Arabidopsis, FvM4K1 was confirmed as a functional Ste20 kinase. Its involvement in the Hippo signalling pathway was further verified through interaction and phosphorylation assays with the two newly identified FvMOB1A and FvMOB1B, homologues of the core component MOB1 in this pathway. Additionally, FvM4K1‐RNAi knockdown and overexpression (OE) lines were generated and analysed, revealing that FvM4K1 positively regulates organ and fruit size in woodland strawberry.

## 2 Results

### 2.1 FvM4K1 Contains the Conserved Ste20 KD

**FIGURE 1:**
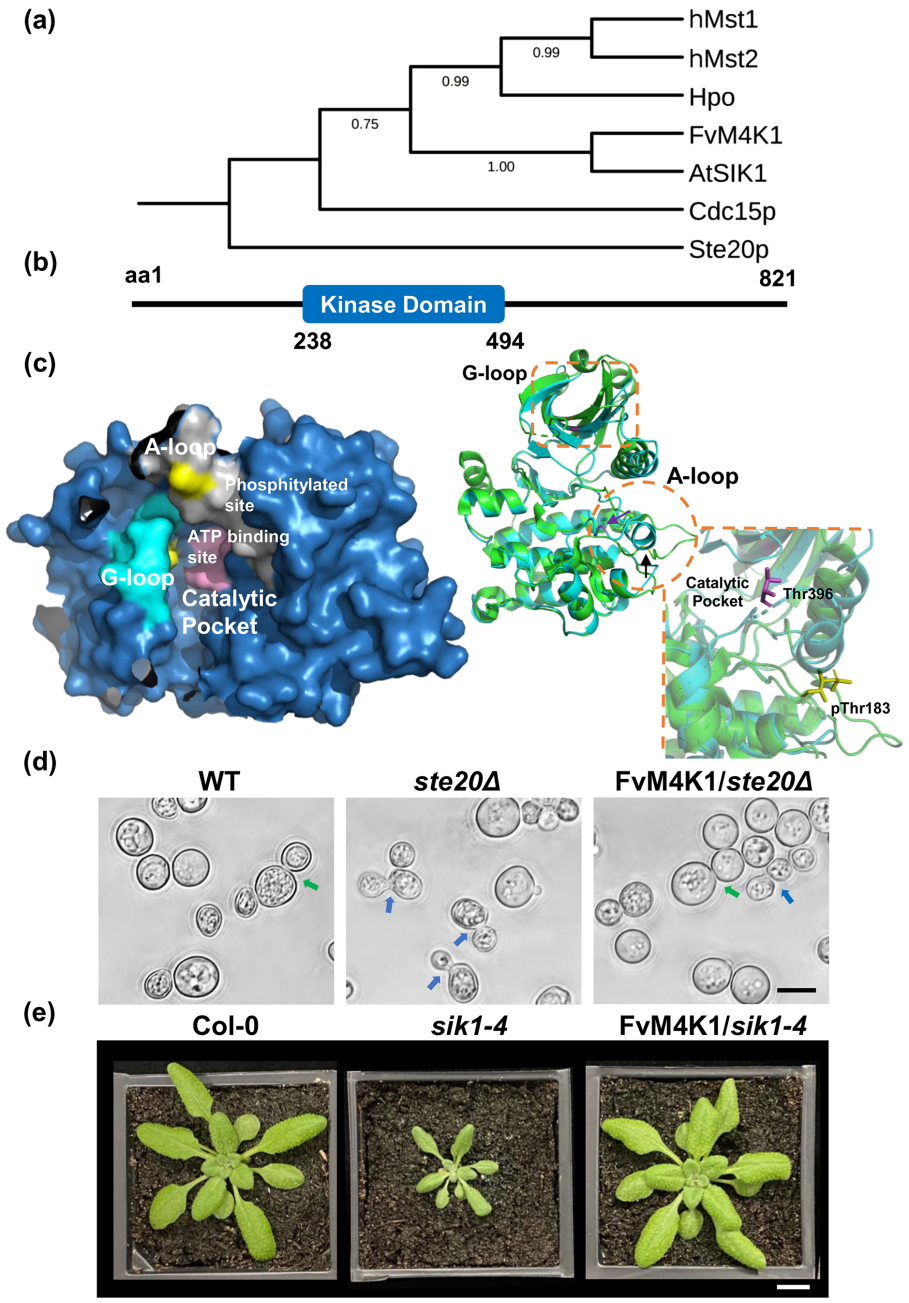
Identification and functional verification of FvM4K1 from woodland strawberry ( *Fragaria vesca* ). (a) Phylogenetic tree created by MEGA7.0 from sequence alignment of kinase domains of FvM4K1 of woodland strawberry, AtSIK1 of Arabidopsis, Ste20p and Cdc15p of yeast, Hpo of Drosophila, and hMst1/2 of humans. (b) Schematic structure of FvM4K1. The kinase domain between aa238–494 is in a blue box. Numbers indicate amino acid positions. (c) Three‐dimensional protein structure prediction by AlphaFold. Left panel, the kinase domain of FvM4K1. The G‐loop (cyan) and the conserved residue Lys269 for kinase activity close to the G‐loop (yellow), the A‐loop (grey) and Thr396 (yellow), the auto‐p site in the A‐loop, the catalytic pocket in the central core and the ATP‐binding site (lilac) within it are indicated. Right panel, alignment between the kinase domains of FvM4K1 and phosphorylated Mst1 (3COM in PDB). Green, phosphorylated Mst1; cyan, non‐phosphorylated FvM4K1. The A‐loop (orange circle) and G‐loop (orange box) are highlighted. Lilac line and purple arrow, Thr396 of FvM4K1; yellow line and black arrow, Thr183 of Mst1. (d) FvM4K1 rescues the budding defect of *ste20Δ* mutant. Yeast cells of WT (BY4741), mutant *ste20Δ* and transgenic *ste20Δ* containing FvM4K1 were observed by phase‐contrast microscopy. Arrows indicate the budding sites for WT (green) and mutant (blue). Scale bar = 5 μm. (e) FvM4K1 rescues the growth defect of *atsik1‐4*, an AtSIK1 loss‐of‐function T‐DNA insertion mutant. Four‐week‐old WT, *sik1‐4*, and transgenic *sik1‐4* expressing FvM4K1. Scale bar = 10 mm.

The genome of woodland strawberry ( *F. vesca* , ‘Hawaii 4’) was searched using BLAST with the amino acid sequence of the KD of Arabidopsis AtSIK1 (At1g69220), a Ste20 homologue (Xiong et al. 2016), resulting in the identification of FvM4K1 (FvH4_4g29800). A maximum likelihood (ML) phylogenetic tree was subsequently constructed using the amino acid sequences of the KDs of FvM4K1 and other known Ste20p homologues, including Cdc15p (YAR019C, P27636) in yeast (*S. cerevisiae*), Hpo (Hippo, Q8T0S6) in fruit fly ( *D. melanogaster* ), hMst1 and hMst2 (Mammalian Ste20‐like kinase 1/2, Q13043, Q13188) in humans, and AtSIK1 in Arabidopsis. FvM4K1 is in the same branch as AtSIK1 and both clustered with Hpo, Mst1 and Mst2, while Cdc15p and Ste20p from yeast are distantly related to FvM4K1 (Figure 1a).

Like Ste20 kinases from yeast and animals, FvM4K1 also contains the conserved KDs. However, notably, the positions of KDs vary significantly between these kinases (Figure S1a). While Ste20 has a C‐terminal KD, Cdc15p, Hpo and Mst1&2 have N‐terminal KDs. In contrast, FvM4K1 (spanning amino acids 238–494, Figure 1b) and AtSIK1 feature KDs positioned one‐third away from the N‐terminus and two‐thirds away from the C‐terminus, suggesting that plant Ste20‐like proteins may function differently compared to their yeast and mammalian counterparts (Figure S1a). In addition, 11 major conserved subdomains (I–XI) were identified (black boxes) in the conserved KDs (Figure S1b). Notably, the glycine‐rich loop (G‐loop) in subdomain I, with the consensus sequence‘GXGXXGXX’, is highly conserved across species. Lys34, identified as the kinase activity site (Taylor and Kornev 2011), is located in subdomain II. Subdomain VIII includes the activation loop (A‐loop) characterised by the consensus sequence ‘KRNT(V/F)(I/V)GTPyWMAPEv’ (purple box), which is recognised as the Ste20 family signature sequence (Delpire 2009). Within this domain, Thr161 (red box) is a conserved auto‐phosphorylation site (auto‐p) linked to catalytic activity (Hanks et al. 1988).

AlphaFold 2 prediction of the three‐dimensional structure of the KD of FvM4K1 shows that it contains a typical G‐loop (cyan) and an A‐loop (grey). The conserved residues Lys269, the kinase activity site, are close to the G‐loop while Thr396, the auto‐p site, is in the A‐loop (both highlighted in yellow, left panel, Figure 1c). The catalytic pocket, located in the central core, contains the ATP‐binding site (lilac) (Mu et al. 2022). To explore the function of FvM4K1, we compared its KD structure with that of the activated Mst1 containing the phosphorylated Thr183 (Protein Data Bank [PDB] code: 3COM). Interestingly, both structures are largely overlapped, showing they are highly conserved (right panel, Figure 1c, green, phosphorylated Mst1; cyan, non‐phosphorylated FvM4K1). The only notable difference lies in the A‐loop (orange circle) where the KD of FvM4K1 folds into a helix with Thr396 (lilac line and purple arrow) hidden in the pocket while the phosphorylated Thr183 (yellow line and black arrow) of Mst1 is extended outward and exposed (Shi et al. 2015).

Therefore, analysis of the KD of FvM4K1 predicted it to be a member of the Ste20 family with a conserved functional KD.

### 2.2 FvM4K1 Functions as Ste20 Family Protein

To determine whether FvM4K1 is a homologue of yeast Ste20p, we transformed the Ste20p lacking mutant *ste20Δ* with FvM4K1. As shown in Figure 1d, the mutant exhibited defects in budding site selection (blue arrows) compared to the WT cells (green arrow) (Sheu et al. 2000). However, when FvM4K1 was present, approximately 70% of the transgenic cells (FvM4K1/*ste20Δ*) displayed a normal polar budding pattern similar to WT (green arrow). This partial rescue of the budding defect phenotype in *ste20Δ* suggests that FvM4K1 is functionally homologous to Ste20p in yeast.

We then investigated whether FvM4K1 functions similarly to AtSIK1, a Ste20 homologue in Arabidopsis (Xiong et al. 2016). To test this, we transformed the Arabidopsis *atsik1‐4* mutant, which lacks *AtSIK1*, with *FvM4K1*. As shown in Figure 1e and Figure S2, the *atsik1‐4* mutant displayed a dwarfed phenotype, with smaller leaves and shorter roots compared to WT (Xiong et al. 2016). However, *atsik1‐4* plants expressing *FvM4K1* were indistinguishable from WT at both the seedling and mature stages, indicating that FvM4K1 fully rescues the growth defects of *atsik1‐4* and thus functions like AtSIK1.

### 2.3 M4K1‐RNAi Knock‐Down Strawberry Plants Showed Smaller Vegetative and Reproductive Organs

**FIGURE 2:**
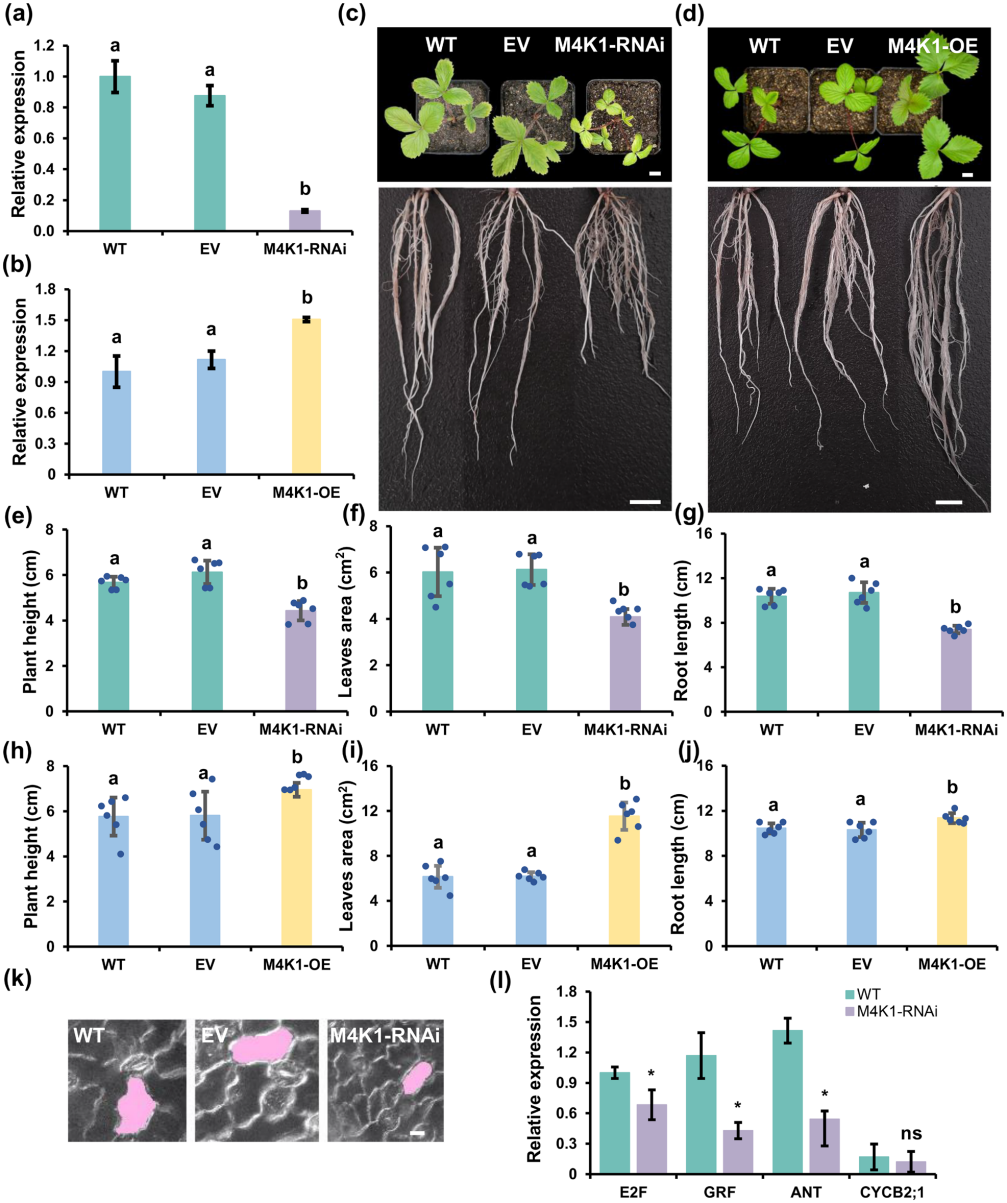
FvM4K1 regulates vegetative organ size in woodland strawberry ( *Fragaria vesca* ). (a) Detection by RT‐qPCR of the transcript level of FvM4K1 in the leaves of 2‐week‐old WT, transgenics harbouring the empty vector (EV) and M4K1‐RNAi plants. (b) Detection by RT‐qPCR of the transcript level of FvM4K1 in the leaves of 2‐week‐old WT, transgenics harbouring the empty vector (EV) plants, and 35S:M4K1 OE plants originated from T0 stolons. The relative transcript level of FvM4K1 was calculated by 2^−∆∆Ct^ method using *FvActin* (FvH4_7g22410) as the reference gene and the transcript level of WT as 1. Error bars = means ± SD (*n* = 3). The different letters indicate a significant difference between samples at *p* < 0.05, calculated by one‐way ANOVA with Duncan test using software SPSS. (c) 8‐week‐old WT, EV and M4K1‐RNAi transgenic strawberry plants. Scale bar = 10 mm. (d) 8‐week‐old WT, EV, and 35S:M4K1 OE strawberry plants originated from T0 stolons. Scale bar = 10 mm. (e–g) Measurements of plant height, leaf area, and root length of WT, EV, and M4K1‐RNAi in (c). (h–j) Measurements of plant height, leaf area, and root length of WT, EV, and M4K1‐OE in (d). Error bars are means ± SD (*n* = 6) in (e–j). Different letters indicate statistically significant differences between RNAi, WT, and EV plants at *p* < 0.05 calculated by one‐way ANOVA with Duncan test using software SPSS. (k) Lower epidermal cells of leaves. A representative cell from each genotype is shaded with pink. Scale bar = 10 μm. (l) Detection by RT‐qPCR of the transcript level of core cell cycle marker genes and regulators in 14‐day‐old seedlings of WT and transgenic M4K1‐RNAi plants. E2F, G1/S specific marker; CYCA2;1, S/G2 specific marker; GRF and ANT, transcription factors that regulate cell size and cell number. The relative transcript level was calculated by the 2^−ΔΔCt^ method using *FvActin* (FvH4_7g22410) as a reference gene and the transcript level of *E2F* in WT as 1. Error bars = means ± SD (*n* = 3). *A difference between samples at *p* < 0.05 in the *t*‐test. ns, no significant difference.

**FIGURE 3:**
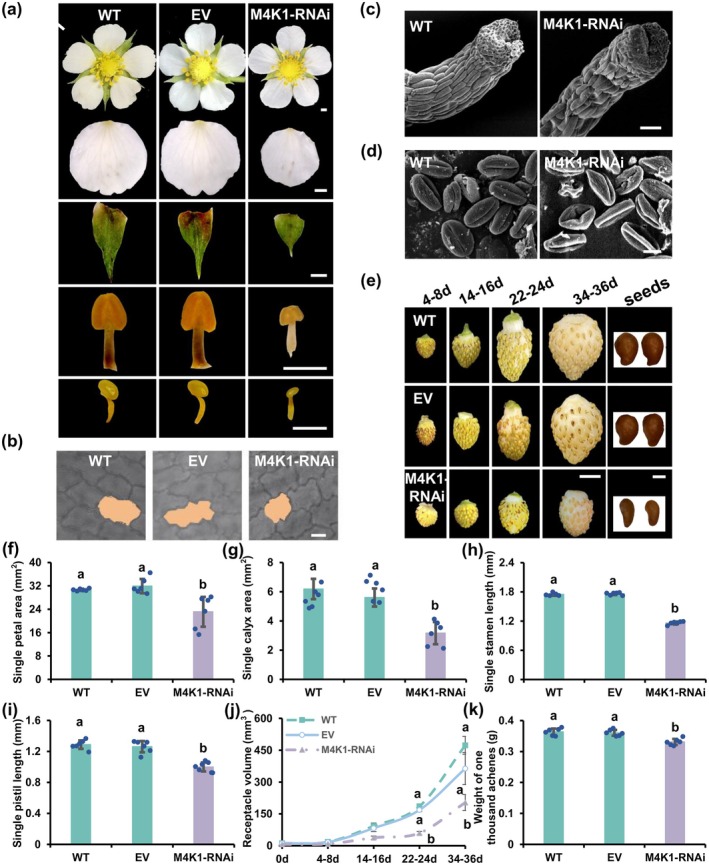
FvM4K1 regulates reproductive organ size of woodland strawberry ( *Fragaria vesca* ). (a) Fully open flowers, petals, calyx, stamen, and pistil from WT, EV, and M4K1‐RNAi. Scale bar = 1 mm. (b) The epidermal cells of petals from WT, EV, and M4K1_RNAi plants. A representative cell from each genotype is shaded with orange. Bar = 10 μm. (c) Scanning electron microscope (SEM) observation of styles. Scale bar = 50 μm. (d) Pollen grains by SEM. Scale bar = 10 μm. (e) Fruits (receptacles) at different developmental stages (days after flowering) and seeds (achenes). Scale bar = 5 mm in the left panel and 0.5 mm in the right panel. (f–i) Measurements of petal area, calyx area, stamen length, and pistil length. (j, k) Measurements of the volume of the receptacles (fruits) at different developmental stages and weight of thousand achenes in mature achenes (seeds). Error bars are means ± SD (*n* = 6). Different letters indicate a statistically significant difference between RNAi, WT, and EV plants at *p* < 0.05 calculated by one‐way ANOVA with Duncan test using software SPSS.

To investigate the biological function of FvM4K1 in woodland strawberry, we generated eight lines of M4K1‐RNAi transgenic strawberry plants (Figure S3a). Downregulation of *FvM4K1* in leaves was confirmed by RT‐qPCR, showing up to 0.8‐fold transcript reduction in six of the eight 2‐week‐old RNAi lines compared to controls (Figure 2a and Figure S3b). We selected line 5 for further phenotypic analysis due to its lowest transcription of *FvM4K1*. The M4K1‐RNAi plants were significantly shorter, with smaller leaves and shorter roots compared to WT (Figure 2c,e–g). Their floral organs, including petals, calyxes, stamens and pistils, were also significantly smaller compared to control plants. Specifically, the petal area and pistil length were reduced by ~23%, and calyx by nearly 50% (Figure 3a,f–i and Figure S3c). The fruits (receptacles) during different developmental stages were also observed, given their importance in marketing value. While no differences were observed between RNAi and WT plants at 4–8 daysafter flowering (DAF) significant differences appeared from 14 to 16 DAF, with the most notable size reduction at 22–24 DAF, where RNAi fruits were only half the size of control fruits (Figure 2e, left panel, j and Figure S3d,e). Additionally, the seeds (achenes) of RNAi fruits were significantly smaller and lighter than controls, although seed length did not show a significant difference (Figure 2e, right panel and Figure S3f,g).

Scanning electron microscopy (SEM) analysis of pollen grains revealed that while WT pollen displayed a regular, plump structure, the majority of pollen grains from M4K1‐RNAi plants appeared shrunken and collapsed (Figure 3d). Abnormalities were also observed in the RNAi pistils, where the style cells were misaligned and many had collapsed compared to the smooth, striated, and regularly aligned style cells in WT plants (Figure 3c). Consistent with this, the RNAi strawberry plants produced fewer seeds, and the majority of the seeds stopped developing at 15 DAF (Figure S8). These findings suggest that FvM4K1 plays a critical role in the development of pollen and pistils during reproduction in strawberry plants.

These results clearly demonstrate that reducing *FvM4K1* expression via RNAi leads to a reduced size in both vegetative and reproductive organs in strawberry plants.

### 2.4 FvM4K1‐RNAi Plants Had Reduced Cell Size and Numbers Compared to WT

We measured the lower epidermal cells of FvM4K1‐RNAi leaves, revealing that they were significantly smaller (630 ± 73 μm^2^, *n* = 10) than those of WT (975 ± 101 μm^2^, *n* = 10) (Figure 2k and Figure S5a). Additionally, the number of pavement cells in M4K1‐RNAi leaves was 629 600 ± 13 026 cells (*n* = 6) compared to 696 966 ± 28 890 in WT (*n* = 6), indicating that the RNAi plants had fewer cells than WT (Figure S5b). Consistent with these results, the cell size and number of petals and seeds (achenes) in RNAi plants were also significantly reduced (Figure S5c,d,h–j).

We further examined the transcript levels of genes associated with the cell cycle (*E2F* and *CYCB2;1*) and the key transcription factors *Growth‐Regulating Factor* (*GRF*) and *AINTEGUMENTA* (*ANT*) in 14‐day‐old seedlings of WT and RNAi plants. As shown in Figure 2l, although the transcript level of *CYCN2;1* was not significantly different, *E2F*, a marker for G1/S and G2/M cell cycle transitions (Desvoyes and Gutierrez 2020) was reduced in RNAi plants compared to WT. Notably, the transcripts of *GRF* and *ANT*, which are positive regulators of cell size and cell number (Mizukami and Fischer 2000; Omidbakhshfard et al. 2015), were downregulated by 0.6‐fold in RNAi compared to control plants. Thus, the expression of genes involved in both cell cycle and its regulation is reduced in the M4K1‐RNAi plants, resulting in a reduction in both cell size and cell number.

### 2.5 FvM4K1‐OE Plants Had Bigger Vegetative and Reproductive Organs With Increased Cell Size and Number

We further generated OE transgenic strawberry plants M4K1‐OE (Figure S4a,b and Figure 2b). Line 4 with the highest *FvM4K1* expression was further phenotyped in the leaves of 2‐week‐old M4K1‐OE strawberry plants originated from T0 stolons, showing that the plants were much larger with bigger leaves and longer roots compared to controls (Figure 2b,h–j). The cell size and number of the lower epidermis of FvM4K1‐OE leaves were significantly increased (Figure S5e–g). The OE fruits at 18–24 DAF were also observed, showing no significant differences between OE and WT plants (Figure S4f,g). The OE plants produced bigger achenes (seeds) than WT (Figure S4c–e). Surprisingly, these bigger seeds failed to germinate (Figure S4h). Therefore, OE of FvM4K1 resulted in overgrowth of cells and lethality in strawberry.

### 2.6 FvM4K1 Is a Kinase and Is Autophosphorylated at Lys269 and Thr396

**FIGURE 4:**
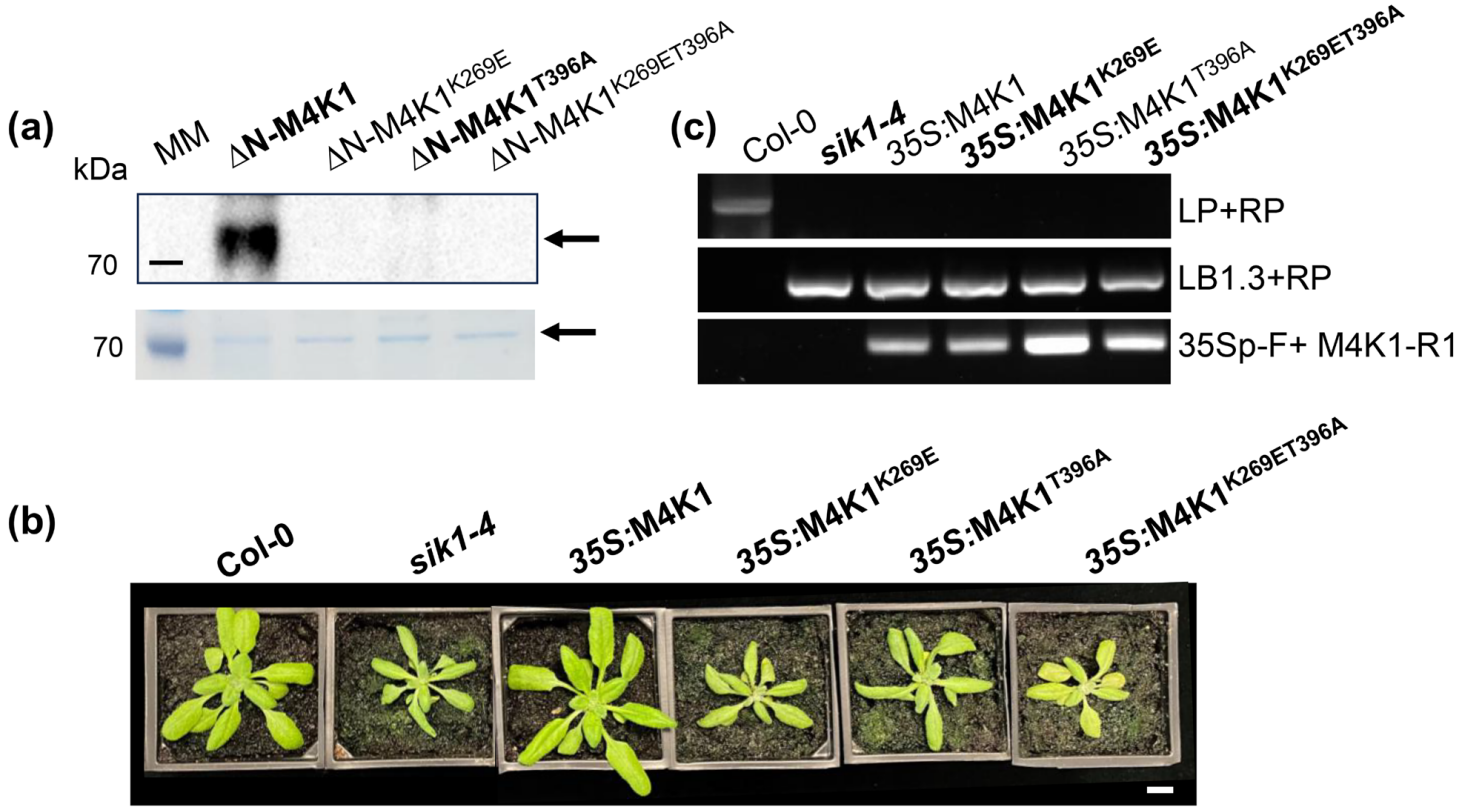
FvM4K1 is autohosphorylated and its kinase activity relies on K269 and T396 for function. (a) In vitro kinase assay. Top panel, Western blot detection with anti‐phosphor‐threonine antibody. A strong band for ∆N‐FvM4K1 was detected while no band was detected in lanes containing the kinase‐dead mutant ∆N‐FvM4K1^K278E^, ∆NFvM4K1^T396A^ and the double mutant ∆N‐FvM4K1^K269ET396A^. Bottom panel, SDS‐PAGE gel stained with EZBlue, showing equal amounts of proteins loaded for ∆N‐FvM4K1 and its pointed mutants. Proteins were expressed in BL21(DB3) and purified. Arrows indicate the positions of FvM4K1 and its variants. MM, protein molecular marker. (b) Four‐week‐old Arabidopsis plants of Col‐0, *sik1‐4* and transgenic *sik1‐4* expressing FvM4K1, FvM4K1^K269E^, FvM4K1^T396A^ and FvM4K1^K269ET396A^. Scale bar = 10 mm. (c) Genotyping by PCR to confirm *sik1‐4* background and the presence of *FvM4K1* in the transgenic plants. Primers sik1‐4‐LP, sik1‐4‐RP, LBb1.3, 35Sp‐F, and M4K1‐R1 are listed in Table S1.

Members of the Ste20 family are known to undergo autophosphorylation (Glantschnig et al. 2002; Wu et al. 2003). To determine whether FvM4K1 is also autophosphorylated and at which sites, we performed an in vitro kinase assay. We expressed a truncated ∆NFvM4K1, lacking the N‐terminus, because the full‐length FvM4K1 could not be expressed in the *E. coli* strain BL21(DE3) (Figure S6a). Additionally, we expressed a kinase‐dead mutant, ∆NFvM4K1^K269E^ as Lys269 is the predicted conserved ATP‐binding site (Figure 1c). The anti‐phospho‐threonine (anti‐pT) antibody detected phosphorylation in ∆NFvM4K1, but not in ∆NFvM4K1^K269E^ (Figure 4a). Thr396 of FvM4K1 within the A‐loop is conserved in Hpo of Drosophila and Mst1/2 of mammals, and this site is the auto‐phosphorylation site (Figure 1c) (Deng et al. 2013; Ni et al. 2013; Praskova et al. 2004). Therefore, we mutated Thr396 to Ala (T396A) and generated ∆NFvM4K1^T396A^. We also combined both mutations and generated the double mutant ∆NFvM4K1^K278ET396A^. The in vitro kinase assay showed that ∆NFvM4K1^T396A^ displayed very weak auto‐phosphorylation activity (weak band, Figure 4a) while no phosphorylation was detected in the double mutant ∆NFvM4K1^K269ET396A^ (no band, Figure 4a). These results clearly demonstrated that FvM4K1, a threonine kinase, both Thr396 and Lys269 play essential roles in phosphorylation of FvM4K1.

### 2.7 Kinase Activity of FvM4K1 Is Essential for Organ Size Control

To validate the in vitro kinase assay results we transformed FvM4K1 and its single and double mutants FvM4K1^K269E^, FvM4K1^T396A^ and FvM4K1^K269ET396A^ in the Arabidopsis *atsik1‐4* mutant. While FvM4K1 fully rescued the growth defects of *atsik1‐4*, mutants expressing FvM4K1^K269E^ and FvM4K1^K269ET396A^ were indistinguishable from the *atsik1‐4* mutant, showing no rescue of growth (Figure 4b and Figure S6b–d). Interestingly, FvM4K1^T396A^ provided partial rescue during early stages of growth as indicated by the larger rosette diameter at 4‐week‐old plants. However, the effect was diminished in older plants, and the 9‐week‐old plants exhibited a similar phenotype as *atsik1‐4*, indicating a loss of rescue over time (Figure 4b and Figure S6b–d).

Therefore, FvM4K1 is a functional kinase; its kinase activity and autophosphorylation rely on Lys269 and Thr396, respectively. Importantly, both kinase activity and autophosphorylation are essential for the role of FvM4K1 in regulating organ size.

### 2.8 FvM4K1 Interacts With FvMOB1A and FvMOB1B, the Core Component of the Hippo Pathway

**FIGURE 5:**
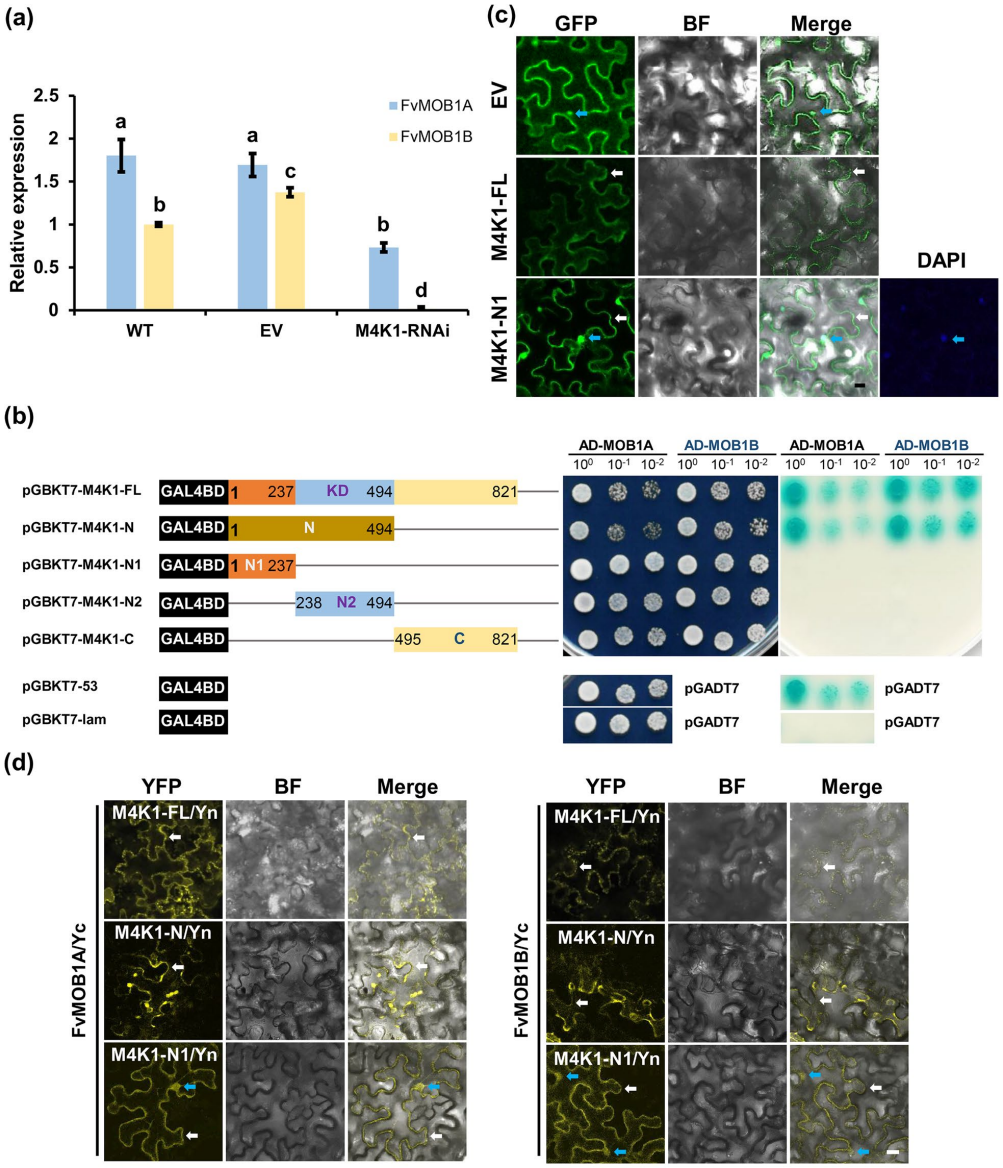
FvM4K1 interacts with FvMOB1A and FvMOB1B. (a) The transcript of *FvMOB1A* and *FvMOB1B* is downregulated in the M4K1‐RNAi strawberry plants. The relative transcript levels were calculated by the 2^−∆∆Ct^ method using *FvActin* (FvH4_7g22410) as the reference gene and expression level of *FvMOB1B* in WT as 1. Error bars = means ± SD (*n* = 3). The different letters indicate significant difference between samples at *p* < 0.05 calculated by one‐way ANOVA with Duncan test using SPSS. (b) Y2H. Left panel, schematic structure of the FvM4K1 gene and fragments; Middle panel, colonies grew on the selective media lacking Trp and Leu (SD/−T/−L); right panel, colonies grew on media lacking Trp, Leu, His, and Ala and supplemented with 200 ng/mL of Aureobasidin A (AbA) and 40 μg/mL of X‐α‐Gal (SD/−T/−L/−H/−A/AbA/X‐α‐Gal). All colonies were grown to reach OD600 = 1; dilutions of 1/10, 1/100 were spotted on the plates. pGBKT7‐53 and pGADT7‐T were positive control pairs while pGBKT7‐lam and pGADT7‐T were negative controls. KD, kinase domain. (c) Subcellular localisation of FvM4K1‐FL and ‐N1. FvM4K1‐FL localisation was detected on the PM while FvM4K1‐N1 was both on the PM and in the nucleus. Blue arrows indicate nucleus, white arrows indicate PM. Scale bar = 20 μm. (d) BiFC. Top panel, FvMOB1A‐YFPc and FvM4K1‐ and its fragments‐YFPn; Bottom panel, FvMOB1B‐YFPc and FvM4K1‐ and its fragments‐YFPn. Blue arrows indicate nucleus, white arrows indicate PM. Scale bar = 20 μm.

In addition to the Ste20 kinase, Mats/Mob1 has been identified as a key component of the Hippo pathway to regulate organ size in both flies and mammals (Praskova et al. 2008; Wu et al. 2003). Homologues of Mob1/Mats have also been found in Arabidopsis, i.e., AtMOB1A (AT5G45550) and AtMOB1B (AT4G19045) (Xiong et al. 2016). Using the protein sequences of AtMOB1A&B to search the strawberry genome, we identified two homologues, FvMOB1A (FvH4_5g23830) and FvMOB1B (FvH4_1g08580) (Figure S7b). Transcription of *FvMOB1A* was reduced by 60% while *FvMOB1B* was undetectable in the FvM4K1‐RNAi plants (line 5) (Figure 5a), suggesting that both FvMOB1A and FvMOB1B function in an FvM4K1‐dependent manner.

To see if FvM4K1 interacts with FvMOB1A&B, we conducted a yeast two‐hybrid (Y2H) assay. The suitability of Y2H was confirmed by a self‐activation test, as no colonies grew on SD/−T/−L/−A/AbA media (Figure S7a). In the experimental assay, colonies harbouring protein pairs between FvM4K1 and FvMOB1s grew well on nutrient‐deficient SD/−T/−L media, indicating gene expression (Figure 5b and Figure S7c). Colonies expressing FvM4K1‐FL/FvMOB1A, FvM4K1‐FL/FvMOB1B and FvM4K1‐N/FvMOB1s on selective media (SD/−T/−L/‐H/−A/AbA/X‐α‐Gal) grew well and turned blue, while those containing FvM4K1‐N1, ‐N2 or ‐C with FvMOB1s struggled to grow and did not turn blue regardless of whether the proteins were fused to AD or BD (Figure 5b and Figure S7c). This indicates that FvM4K1 interacts with FvMOB1A and FvMOB1B, and this interaction depends on an intact KD within the N‐terminus of FvM4K1.

### 2.9 Interactions Between FvM4K1 and FvMOB1s Occur at Both the Plasma Membrane and Nucleus, Which Require an Intact FvM4K1 N‐Terminus

The interaction between FvM4K1 and FvMOB1 proteins was further confirmed in tobacco leaves using bimolecular fluorescence complementation (BiFC). As shown in Figure 5d and Figure S7d, yellow fluorescent protein (YFP) signals were detected at the plasma membrane (PM; white arrows) when FvM4K1‐FL was co‐expressed with either FvMOB1A or FvMOB1B, as well as when FvM4K1‐N was co‐expressed with FvMOB1A or FvMOB1B, indicating interactions at the PM. Notably, YFP signals were also observed at both the PM and nucleus (blue arrows) when FvM4K1‐N1 was co‐expressed with FvMOB1A or FvMOB1B, suggesting that nuclear localisation of FvM4K1 occurs independent of its KD.

We further assessed the subcellular localisation of FvM4K1 and FvM4K1‐N1 by expressing their GFP fusions in tobacco leaves. Confocal microscopy revealed that FvM4K1 localises to the PM (Figure 5c), while FvM4K1‐N1‐GFP was observed both at the PM (white arrows) and in the nucleus (blue arrows, confirmed by DAPI staining). These results suggest that FvM4K1 localises to the PM, which is crucial for its interaction with FvMOB1 proteins.

### 2.10 FvM4K1 Phosphorylates FvMOB1A and FvMOB1B

**FIGURE 6:**
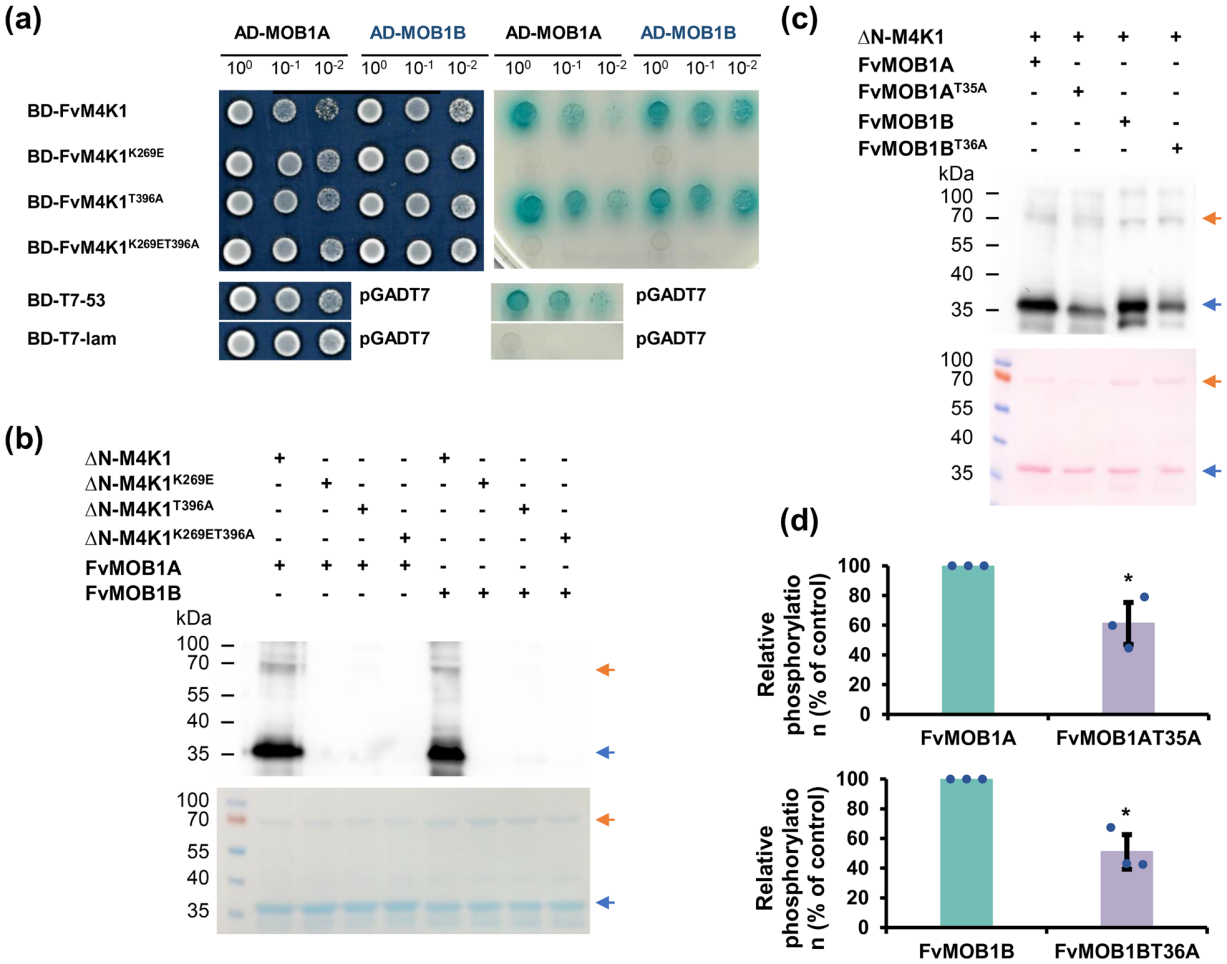
Interaction between FvM4K1 and FvMOB1A&B relies on the kinase activity of FvM4K1. (a) Yeast two‐hybrid assay. Left panel, yeast colonies on SD/−T/−L selective media showing yeast growth; right panel, yeast colonies on SD/−T/−L/−H/−A/AbA/X‐α‐Gal showing interaction. Aureobasidin A (AbA, 200 ng/mL) and X‐α‐Gal (40 μg/mL) were added to the media. All colonies were grown to reach OD600 = 1; dilutions of 1/10 and 1/100 were spotted on the plates. pGBKT7‐53 and pGADT7‐T were positive controls while pGBKT7‐lam and pGADT7‐T were negative control pairs. (b) FvMOB1A and FvMOB1B are phosphorylated by FvM4K1, detected by in vitro kinase assay. Top panel, detection by Western blot with anti‐phosphor‐threonine antibody; lower panel, EZBlue stained gel. Orange arrows indicate the positions of ∆NFvM4K1 and blue arrows FvMOB1A and FvMOB1B. All proteins were expressed in BL21(DB3) and purified. (c) FvMOB1A and FvMOB1B are phosphorylated at Thr35 and Thr36, respectively, determined by in vitro kinase assay. Top panel, detection by Western blot with anti‐phosphor‐threonine antibody; bottom panel, Ponceau S stained blot. Orange arrows indicate the positions of ∆NFvM4K1 and blue arrows FvMOB1A and FvMOB1B. All proteins were expressed in BL21(DB3) and purified. (d) Quantification of phosphorylation of WT and mutants of FvMOB1A and FvMOB1B in (c). The phosphorylation (band intensity) of FvMOB1A and FvMOB1B on the blot was regarded as 100%. The phosphorylation of mutant AtMOB1s^TA^ was calculated as the percentage of the peak intensity of AtMOB1s. Error bars are means ± SD (*n* = 3). *The difference between samples at *p* < 0.05 in the *t*‐test.

Since Ste20 family kinases, like Hpo and Mst1/2, are known to phosphorylate their interacting partners, it is likely that FvM4K1 also phosphorylates FvMOB1A and FvMOB1B. To test this, we conducted Y2H and in vitro kinase assays. After ruling out false positives via the Y2H self‐activation test (Figure S7a), we observed colony growth on nutrient‐deficient SD/−T/−L media (Figure 6a, left panel). However, on selective media (SD/−T/−L/‐H/−A/AbA/X‐α‐Gal), colonies expressing combinations of FvM4K1^T396A^/FvMOB1A and FvM4K1^T396A^/FvMOB1B grew well and turned blue, similar to pairs between FvM4K1‐FL and FvMOB1s. However, colonies expressing FvM4K1^K269ET396A^/FvMOB1A and FvM4K1^K269ET396A^/FvMOB1B did not grow or turn blue (Figure 6a, right panel). These findings indicate that a mutation in FvM4K1^K269E^ disrupts interaction with FvMOB1s, while FvM4K1^T396A^ retains its interaction capability. Thus, the interaction between FvM4K1 and FvMOB1s depends on Lys269, linked to kinase activity, rather than Thr396 in the A‐loop for autophosphorylation.

To directly demonstrate the role of phosphorylation in the interaction, we expressed and purified the relevant proteins and conducted an in vitro kinase assay. As shown in Figure 6b, both FvMOB1A and FvMOB1B were detected with an anti‐phospho‐threonine antibody (anti‐pT) (arrows in the first and fifth lanes) when ΔNFvM4K1 was present, confirming that FvM4K1 phosphorylates both FvMOB1A and FvMOB1B (top panel). In contrast, only faint or no bands were observed for FvMOB1A and FvMOB1B when FvM4K1^T396A^ or FvM4K1^K269E^ were used, indicating these mutants had little or no ability to phosphorylate FvMOB1s.

These findings demonstrate that the interaction between FvM4K1 and FvMOB1s depends on both trans‐ and autophosphorylation of FvM4K1.

### 2.11 Identification of Phosphorylation Sites in FvMOB1A and FvMOB1B by FvM4K1

To identify the specific amino acid residues in FvMOB1A and FvMOB1B that were phosphorylated by FvM4K1, we looked into previous studies on hMob1, which identified Thr12 and Thr35 as phosphorylation sites, with Thr35 being more impactful (Praskova et al. 2008). Notably, Thr35 is conserved in FvMOB1A and as Thr36 in FvMOB1B (Figure S7b). We therefore mutated these residues to alanine (FvMOB1A^T35A^ and FvMOB1B^T36A^) and expressed the mutant proteins in *E. coli* (Figure 6c, bottom panel). After the kinase reactions where 200 ng of FvM4K1 and 1 μg of FvMOB1A, FvMOB1A^T35A^, FvMOB1B, or FvMOB1B^T36A^ were added, all four FvMOB1s could be detected by Western blot with anti‐pT antibody. However, phosphorylation of FvMOB1A^T35A^ and FvMOB1B^T36A^ was reduced by 40% and 50% compared to their wild‐type counterparts (Figure 6c, top panel, Figure 6d). Thus, FvM4K1 phosphorylates both FvMOB1A and FvMOB1B, with Thr35 in FvMOB1A and Thr36 in FvMOB1B as key phosphorylation sites.

## 3 Discussion

### 3.1 FvM4K1 Functions as a Typical Ste20‐Like Protein in Woodland Strawberry

We identified FvM4K1 (FvH4_4g29800), a Ste20‐like protein, in woodland strawberry ( *Fragaria vesca* , ‘Hawaii 4’) (Figure 1). FvM4K1 rescued the growth defect of a yeast *ste20Δ* mutant lacking Ste20p (Figure 1d), similar to how Arabidopsis AtSIK1 can restore the same mutant (Xiong et al. 2016). This suggests that plant Ste20‐like proteins, such as FvM4K1 and AtSIK1, retain the conserved function of Ste20p in regulating organ size via cell division. The role of Ste20 family kinases in controlling organ size through the Hippo pathway is well established in flies and mammals (Lu, Li, et al. 2010; Wu et al. 2003). In humans, Ste20‐like proteins Mst1/2 auto‐phosphorylate and trans‐phosphorylate substrates at serine and threonine residues (Creasy and Chernoff 1995a, 1995b). While AtSIK1, a Ste20 homologue in Arabidopsis, has been shown to autophosphorylate, its ability to trans‐phosphorylate substrates has not been reported (Zhang et al. 2018).

To determine whether FvM4K1 undergoes autophosphorylation, we performed in vitro kinase assays. Detection with an anti‐phospho‐threonine antibody confirmed FvM4K1 autophosphorylation, establishing it as a threonine kinase (Figure 4a). Lys269 within the conserved ATP‐binding site is conserved with other Ste20 kinases (Figure 1c). Phosphorylation is absent in kinase‐dead mutants such as Hpo^K71R^ in Drosophila and Mst1^K59R^ in mice (Glantschnig et al. 2002; Wu et al. 2003), underscoring the importance of this lysine residue in kinase activity. Consistent with this, the ability of FvM4K1^K269E^ to auto‐phosphorylate and trans‐phosphorylate FvMOB1A and FvMOB1B was abolished (Figures 4a and 6b,c), confirming FvM4K1 as a threonine kinase. Thr396 in the A‐loop of FvM4K1 (Figure 1c and Figure S1) is also conserved with Thr183/Thr180 in Mst1/2 and Thr195 in Hpo (Glantschnig et al. 2002; Jin et al. 2012). Mutations at these sites in Mst1/2 significantly reduce phosphorylation (Glantschnig et al. 2002; Ni et al. 2013; Praskova et al. 2008). Similarly, we found that both auto‐phosphorylation and trans‐phosphorylation activities were abolished in the FvM4K1^T396A^ mutant (Figures 4a and 6b,c), demonstrating that autophosphorylation at Thr396 is essential for FvM4K1 function.

Thus, the lysine residue in subdomain II (Lys269) and the threonine residue in the A‐loop (Thr396) are crucial for the kinase activity and autophosphorylation of FvM4K1. These structural features are conserved across Ste20 kinases in plants, yeast, flies and mammals, although their positions are different due to the positions of the KDs in these proteins (Figure 1c and Figure S1).

### 3.2 The Role of the Hippo Pathway in Strawberry: Kinase‐Scaffold Interactions and Phosphorylation Dynamics in Plants

The Hippo pathway is a critical signalling mechanism that regulates organ size by controlling kinase‐scaffold protein interactions and phosphorylation events. A Ste20 kinase interacts with a WW‐domain scaffold protein, leading to phosphorylation of downstream complexes, including Mob1 family proteins and LAT/NDR kinases, which in turn regulate target proteins involved in organ size control (Zhao et al. 2011; Zheng and Pan 2019). While well‐characterised in Drosophila and mammals, the core components of the Hippo pathway in plants are less understood. In Arabidopsis, three components have been identified, AtSIK1 (Ste20 homologue), AtMOB1A/B (Mob1 homologues) and eight AtNDRs (LAT/NDR homologues) (Guo et al. 2020; Xiong et al. 2016; Yoon et al. 2021). Both AtMOB1A and AtMOB1B interact with AtSIK1 and NDR2/4/5, similar to the conserved interactions found in animals (Praskova et al. 2008; Wei et al. 2007). This suggests that the Hippo pathway shares conserved components and interactions between plants and animals.

The AtSIK1‐AtMOB1A/B interactions have been confirmed in Arabidopsis; however, the nature of this interaction remains unknown (Xiong et al. 2016). To address this, we performed yeast two‐hybrid assays and demonstrated that their homologues, FvM4K1 and FvMOB1A/B in woodland strawberry, also interact. This interaction depends on the intact N‐terminus of FvM4K1, as neither the N1 fragment (lacking the KD) nor the N2 fragment (containing only the KD) interacted with FvMOB1s (Figure 5b and Figure S7c). We further validated the interactions using bimolecular fluorescence complementation assays in tobacco, although a discrepancy emerged showing an unexpected interaction between FvM4K1‐N1 and FvMOB1s (Figure 5d). Importantly, the interaction between FvM4K1 and FvMOB1s is kinase‐dependent, as no interaction was observed between the kinase‐dead mutant FvM4K1^K269E^ and FvMOB1s in Y2H assays (Figure 6a). This was further corroborated by in vitro kinase assays where FvMOB1A and FvMOB1B were phosphorylated by FvM4K1. A mutation at Thr396 in FvM4K1 significantly reduced its ability to phosphorylate FvMOB1s (Figure 6b), indicating that the kinase activity of FvM4K1 is self‐regulated through autophosphorylation.

In humans, Mst1 phosphorylates hMob1A at two key sites, Thr12 and Thr35, with Thr35 having a more profound effect on Mob1A phosphorylation (Praskova et al. 2004). Similarly, phosphorylation of FvMOB1A^T35A^ and FvMOB1B^T36A^ was reduced by up to 50% (Figure 6c,d), suggesting that these threonine residues are critical phosphorylation sites. Interestingly, the auto‐phosphorylated Mst2 binds hMob1A and its mutant hMob1A^T12A/T35A^ more effectively than inactive Mst2. However, in the presence of ATP and Mg^2+^, Mst2 binding to hMob1A decreased significantly, while the binding to hMob1A^T12A/T35A^ remained unaffected (Praskova et al. 2008). This suggests that phosphorylation of hMob1A by Mst2 promotes complex dissociation. A similar dynamic may occur between FvM4K1 and FvMOB1s, where phosphorylation is critical for interaction, but other structural elements likely contribute to the stability of the interaction complex. This, of course, remains to be determined.

### 3.3 Divergent Roles of the Hippo Pathway in Organ Size Regulation: FvM4K1‐Mediated Growth in Strawberry and Functional Comparisons With Animal Systems

To investigate the role of FvM4K1 in woodland strawberry, we generated FvM4K1‐RNAi knockdown plants. One RNAi line (line 5) with significantly reduced *FvM4K1* expression exhibited reduced stature with smaller vegetative and reproductive organs, which resulted from a reduction in both cell size and cell number (Figures 2 and 3; Figures S3 and S5). In contrast, plants overexpressing FvM4K1 (M4K1‐OE) displayed increased height and larger leaves, associated with increased cell size and number (Figures 2d,h–j and 5; Figure S4). These results demonstrate that FvM4K1 regulates organ size in strawberry by promoting cell expansion and proliferation. Similarly, Arabidopsis mutants, such as *atsik1‐4*, lacking AtSIK1, show stunted growth with smaller organs caused by reduced cell size and number compared to wild‐type plants (Xiong et al. 2016). Strikingly, heterologous expression of FvM4K1 in *atsik1‐4* rescued these defects, whereas kinase‐dead (FvM4K1^K269E^) or phospho‐threonine mutant (FvM4K1^T396A^) variants did not (Figure 1e and Figure S2). This confirms functional conservation between FvM4K1 and AtSIK1, both acting as positive regulators of plant growth. This contrasts with the role of Ste20 family kinases in animals, where loss‐of‐function mutations often result in overgrowth. For example, in fruit fly, mutations in Hpo kinase lead to tissue overgrowth, resulting in larger organs such as larger eyes, due to excessive cell proliferation (Wu et al. 2003). Similarly, overexpressing Hpo phosphorylation mutants T195A or kinase‐dead K71R causes enlarged wings while wild‐type flies exhibit smaller and more compact wings (Jin et al. 2012). Thus, in animals, Ste20 family kinases act as negative regulators of cell proliferation.

Ste20 kinases are key components of the Hippo signalling pathway, which regulates organ size through interactions with and phosphorylation of other pathway components (Zhao et al. 2011; Zheng and Pan 2019). Consequently, mutations in these components can also result in growth defects similar to those seen in Ste20 mutants. For example, in Drosophila, mutations in *wts* and *sav* lead to tissue overgrowth (Justice et al. 1995; Kango‐Singh et al. 2002), while co‐expression of *Hpo* and *Mats* significantly reduces eye size (Lai et al. 2005; Wu et al. 2003). Conversely, in Arabidopsis, the *mob1a−/− mob1b+/−* mutant, which lacks AtMOB1A on both chromosomes and AtMOB1B on one, shows reduced plant height, shorter roots and siliques (Guo et al. 2020). More severe growth defects are observed in the *sik1−/− mob1a−/−* and *sik1−/− mob1a+/−* double mutants, where growth arrest occurs and seed germination is impaired, indicating growth suppression in these mutants (Xiong et al. 2016). Thus, while the Hippo pathway is evolutionarily conserved across eukaryotes, it appears to play opposite roles in regulating cell proliferation and organ size in plants and animals. In plants, the Hippo pathway promotes growth, whereas in animals, it suppresses growth.

Despite opposing roles in growth regulation, the Hippo pathway appears functionally conserved in reproduction between plants and animals. In *FvM4K1*‐RNAi strawberry plants, some seeds (achenes) failed to develop and aborted (Figure S8), likely due to abnormal pollen grains or pistils observed by SEM (Figure 3c,d). This is consistent with the observation in Arabidopsis where the mutant *atsik1‐4* had fewer seeds per silique than WT, although the cause remains to be explored (Xiong et al. 2016). Interestingly, OE of FvM4K1 in woodland strawberry caused lethality, as T1 achenes from M4K1‐OE plants failed to germinate (Figure S4f). In flies, the functional loss of Hpo in eyes leads to pupal lethality in eyeless flies. Additionally, 66% of *hpo* null mutants die, and none survive when a heterozygous *sav* or *wts* mutation is introduced. Co‐expression of *Hpo* and *Wts* causes complete lethality (Wu et al. 2003). These findings suggest a conserved role for the Hippo pathway in reproduction across species.

**FIGURE 7:**
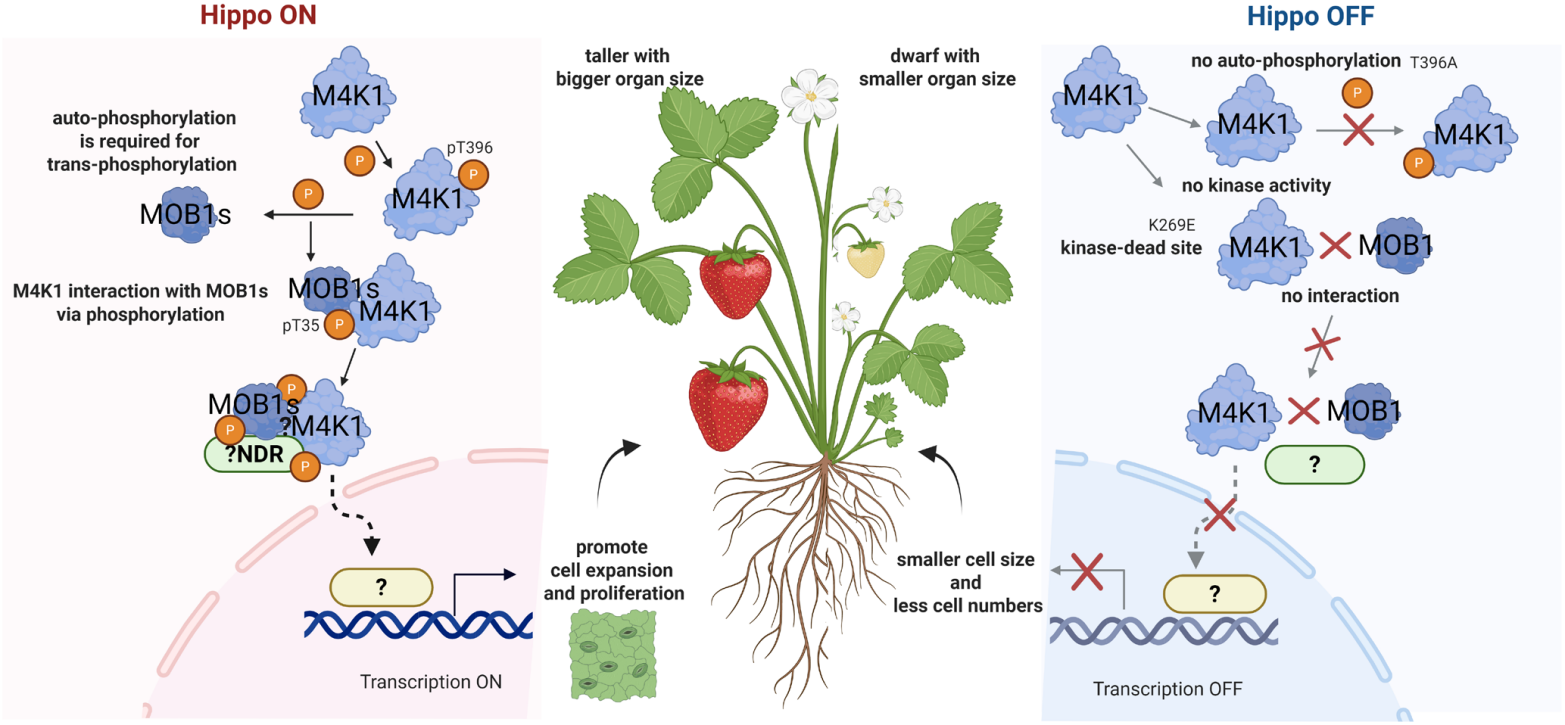
The proposed model of Hippo signalling pathway in organ size control in woodland strawberry ( *Fragaria vesca* ). The FvM4K1 regulates organ size and this depends on its kinase activity. In its active form (Hippo on, left panel), FvM4K1 interacts with and phosphorylates Hippo pathway components, such as MOB1s, a process facilitated by autophosphorylation of FvM4K1 at Thr396. FvM4K1 may also interact with and phosphorylate the NDR family proteins aided by the phosphorylated FvMOB1s. This leads to the activation of transcription factors which enter the nucleus where they can trigger the transcription of genes involved in cell proliferation, resulting in larger strawberry fruits and other organs. However, when this pathway is off (right panel), FvM4K1 is unable to autophosphorylate (T396A) and/or does not have kinase activity (K296E, kinase dead). Therefore, interaction and trans‐phosphorylation of FvMOB1s and other Hippo pathway components cannot be achieved, genes controlling cell size and number are not activated, thus the observed smaller organ size in strawberry.

The plant Ste20‐like kinases AtSIK1 and FvM4K1 have a simplified structure which may alter the kinase interactions. Animal Hpo/Mst1/2 kinases possess both kinase and SARAH domains, the latter enabling interaction with WW‐domain scaffold proteins like Sav (Cairns et al. 2018). In contrast, plant homologues (e.g., FvM4K1, AtSIK1) retain only the KD (Figure 1b and Figure S1a). This structural simplification suggests evolutionary rewiring, i.e., without SARAH domains or Sav homologues (undetected in strawberry or Arabidopsis), plant Ste20 kinases may bypass scaffold‐mediated complex assembly. Instead, they interact directly with MOB1/2 (Figures 5 and 6) and potentially NDR kinases (Zhou et al. 2021), similar to yeast systems where Ste20 kinases function independently of scaffolds (Gruneberg 2000). This autonomy could alter downstream signalling logic, for example, enabling direct phosphorylation of growth‐promoting targets without the inhibitory “brakes” imposed by animal scaffold complexes. This suggests evolutionary streamlining in plants, with AtSIK1 and FvM4K1 functioning autonomously to mediate downstream phosphorylation. In addition, the lack of Yki/YAP homologues, the transcriptional factors central to animal Hippo signalling, further distinguishes plant Hippo pathways. In animals, Yki drives proliferation when Hippo signalling is inactive; its phosphorylation by Lats kinases sequesters it in the cytoplasm, suppressing growth (Zheng and Pan 2019). Plants lack Yki homologues but retain NDR kinases (e.g., AtNDR2/4/5) (Yoon et al. 2021), which may regulate distinct transcription factors to promote cell division and expansion. For instance, Arabidopsis NDR kinases modulate auxin signalling (Zhou et al. 2021), a pathway critical for plant growth. We hypothesise that FvM4K1 phosphorylates NDR kinases to activate TFs regulating cell cycle genes (e.g., *CYCD*; Figure 7) or cell wall modifiers, linking Hippo signalling to plant‐specific growth processes.

The functional inversion of Hippo signalling (growth promotion in plants vs. suppression in animals) likely reflects evolutionary adaptation. Animals require precise control of organ size to maintain body plan symmetry, necessitating pathways that curb proliferation (e.g., via apoptosis). Plants, however, exhibit indeterminate growth and rely heavily on post‐embryonic cell division and expansion. The co‐option of Hippo components to promote growth may align with these demands. Supporting this, Arabidopsis *mob1* mutants display root meristem defects (Guo et al. 2020), underscoring the role of the Hippo pathway in sustaining proliferative tissues, a stark contrast to animal Hippo mutants, where unchecked proliferation causes overgrowth.

Plant Hippo signalling may intersect with hormone pathways absent in animals. For example, AtSIK1 interacts with brassinosteroid (BR) signalling components (Kim et al. 2023), and BRs promote cell elongation, a process amplified in *FvM4K1*‐OE plants (Figure 2h–j). Similarly, crosstalk with auxin or cytokinin pathways could explain the dual regulation of cell division and expansion (Cui et al. 2016; Pinosa et al. 2013; Xiong et al. 2016; Zhang et al. 2021). This integration with phytohormone networks, a hallmark of plant signalling, may have repurposed ancestral Hippo components into growth‐promoting hubs.

Based on our findings in this study, we propose a model for the Hippo pathway in woodland strawberry. FvM4K1 positively regulates organ size and this relies on its kinase activity. In its active form (“Hippo on”, Figure 7, left panel), FvM4K1 undergoes autophosphorylation and interacts with and phosphorylates FvMOB1A and FvMOB1B. FvM4K1 may also interact with and phosphorylate NDR family proteins, leading to the activation of transcription factors and the expression of genes that promote cell proliferation and organ enlargement. However, when the pathway is inactive (“Hippo off”, Figure 7, right panel), FvM4K1 is unable to undergo auto‐phosphorylation or trans‐phosphorylate downstream components, resulting in reduced organ size.

In summary, FvM4K1 functions as a kinase in the Hippo signalling pathway in woodland strawberry by interacting with and phosphorylating the scaffold proteins FvMOB1A and FvMOB1B, homologous to those found in animals. Like AtSIK1 in Arabidopsis, FvM4K1 positively regulates organ size in strawberry, contrasting with the typically negative regulatory role of Ste20 family kinases in animals. Although the molecular basis for this functional divergence remains unclear, the functional divergence of Hippo signalling in plants likely stems from structural simplification (loss of SARAH domains/scaffolds), enabling direct kinase‐substrate interactions, loss of animal‐specific components (Yki, Sav) and acquisition of plant‐specific targets (e.g., NDR kinases, hormone regulators), and evolutionary rewiring to meet the demands of indeterminate growth, prioritising cell division/expansion over apoptosis. Future work defining FvM4K1‐NDR‐TF networks and their crosstalk with hormone pathways may clarify how plants repurposed this ancient pathway.

## 4 Experimental Procedures

### 4.1 Plant Materials

Woodland strawberry ( *Fragaria vesca* , ‘Hawaii 4’) was grown under 11 h‐light (120 μm m^−2^ s^−1^) and 13 h‐dark at 21°C. Wild‐type Arabidopsis (Col‐0) and the T‐DNA insertion lines *atsik1‐4* (SALK_051369) (Xiong et al. 2016) were obtained from the Arabidopsis Biological Resource Centre (ABRC, http://www.arabidopsis.org/abrc/) and grown under a 16 h‐light (120 μm m^−2^ s^−1^) and 8 h‐dark cycle at 21°C.

### 4.2 Bioinformatic Analysis

For phylogenetic analysis, the amino acid sequences of the KD of strawberry FvM4K1 (FvH4_4g29800) were aligned with Ste20p and Cdc15p from yeast (Bardin et al. 2003; Peter et al. 1996), Hpo from fruit fly (Wu et al. 2003), hMst1/2 from humans (Ni et al. 2013; Praskova et al. 2004) and AtSIK1 from Arabidopsis (Xiong et al. 2016) ClustalW in MEGA7.0 software. A ML phylogenetic tree was constructed using the Poisson substitution model and visualised with iTOL (https://itol.embl.de/).

For 3D protein structure prediction, the KD of FvM4K1 was modelled using AlphaFold (https://colab.research.google.com/github/deepmind/alphafold/blob/main/notebooks/AlphaFold.ipynb). The predicted structure with the highest mean pLDDT (88.59) was visualised in PyMOL (Jumper et al. 2021; Varadi et al. 2022) and aligned with the known structure of the Mst1 KD (PDB code: 3COM) to indicate the active conformation of FvM4K1.

### 4.3 Yeast Complementation Assay

The coding region of *FvM4K1* was cloned into the yeast expression vector pYES‐DEST52 using Gateway cloning (Invitrogen) and transformed into wild‐type yeast BY4741 and the *ste20Δ* mutant (Y00956) (MATa, ura3Δ0, leu2Δ0, his3Δ1, met15Δ0, YHL007c::kanMX4) (EUROScarf, http://www.euroscarf.de/). Empty vector controls were also transformed into both yeast strains. Colonies were grown on selective media containing 2% galactose to induce protein expression. Cells were observed using phase‐contrast microscopy (Zeiss LSM 710).

### 4.4 Arabidopsis and Strawberry Transformation

C‐terminal GFP‐tagged FvM4K1 and its mutants (FvM4K1^K269E^, FvM4K1^T396A^, FvM4K1^K269ET396A^) were cloned into pH7FWG2‐293 via Gateway cloning (Katzen 2007; Zhang et al. 2022) and transformed into *atsik1‐4* Arabidopsis using the floral dipping method, followed by genotyping (Qi et al. 2004).

For FvM4K1‐RNAi strawberry plants, a ~200 bp fragment from the KD of FvM4K1 was cloned into pK7GWIWG2(II)‐RR‐277 to generate a hairpin structure (Zhang et al. 2022) and transformed into woodland strawberry. Empty vector controls were also transformed. OE of FvM4K1 was achieved by cloning the full‐length gene into pH7FWG2‐293 (Zhang et al. 2022).

### 4.5 Yeast Two‐Hybrid (Y2H) and Bimolecular Fluorescence Complementation (BiFC) Assays

For Y2H, the full‐length *FvM4K1*, phosphorylated site mutants, and different fragments of *FvM4K1* were cloned into pGBKT7, while *FvMOB1A* and *FvMOB1B* were cloned into pGADT7 (Lu, Tang, et al. 2010). Yeast strain Y2H Gold (Clontech) was used for transformation. Positive and negative control plasmids (pGBKT7‐53/pGADT7‐T and pGBKT7‐Lam/pGADT7‐T) were co‐transformed into Y2H Gold as controls. Yeast cells were cultured in SD/−T/−L liquid media, and 10‐fold serial dilutions were spotted onto selective SD/−T/−L and SD/−T/−L/‐H/−A/AbA/X‐α‐Gal plates for reporter gene expression, and plates were scanned after 4 days.

For BiFC, the same constructs used in Y2H were cloned into pGTQL1221YN/C (Lu, Tang, et al. 2010) and transformed into *Agrobacterium tumefaciens* strain GV3101 for infiltration into tobacco leaves. Confocal microscopy (Zeiss LSM 710) was used to observe fluorescence after infiltration. For DAPI staining, 5 μg/mL of DAPI was infiltrated into the leaves and incubated for 2 h before observation under UV light.

### 4.6 Subcellular Localisation

Full‐length FvM4K1 and FvM4K1‐N1 (lacking the KD) were cloned into pSuper1300 (Liang et al. 2020) to create C‐terminal GFP fusion proteins. These constructs were transiently expressed in tobacco leaves and visualised by confocal microscopy.

### 4.7 Protein Purification and In Vitro Kinase Assay

A truncated version of FvM4K1 lacking the first 236 amino acids (∆N‐FvM4K1) and its mutants (∆N‐FvM4K1^K269E^, ∆N‐FvM4K1^T396A^, ∆N‐FvM4K1^K269ET396A^) were cloned into the *E. coli* expression vector pET‐28 (Novagen) and expressed in BL21(DE3) cells. Protein expression was induced with 0.7 mM IPTG, and proteins were purified using His GraviTrap (Cytiva).

In vitro kinase assays were performed with 200 ng of purified ∆N‐FvM4K1 or mutant proteins for auto‐phosphorylation assays. To assess phosphorylation of FvMOB1 proteins or their mutants, 1000 ng of substrate protein was added to the reaction in kinase buffer (20 mM Tris–HCl, pH 7.5, 10 mM MgCl₂, 100 μM ATP, 10 mM EDTA, 10 mM NaF). The reaction was incubated at 30°C for 30 min, and proteins were resolved on a 10% SDS‐PAGE gel. Phosphorylation was detected by Western blot using a phospho‐threonine antibody (Cell Signalling), followed by ECL detection. Loading controls were assessed via EZBlue staining or Ponceau S staining.

### 4.8 Data Analysis

Statistical analysis was performed using SPSS software. Error bars in the figures represent the standard deviation (SD), and the number of replicates is provided in the figure legends. A two‐tailed *t*‐test was used for statistical comparisons between two samples. For comparisons among multiple samples, one‐way ANOVA followed by Duncan's post hoc test was performed. Statistically significant differences (*p* < 0.05, as indicated in the figure legends) are denoted by different letters (e.g., a, b, c) or an asterisk (*).

### 4.9 Accession Numbers

Sequence data for woodland strawberry in this study are available in the Genome database for Rosaceae (RGD, https://www.rosaceae.org) and Arabidopsis in the Arabidopsis Biological Resource Centre (ABRC, http://www.arabidopsis.org/abrc/). The accession numbers are *FvM4K1* (FvH4_4g29800), *FvMOB1A* (FvH4_5g23830) and *FvMOB1B* (FvH4_1g08580) from RGD, *AtSIK1* (At1g69220), *AtMOB1A* (At5g45550) and *AtMOB1B* (At4g19045) from ABRC, *Ste20p* (Q03497), *Cdc15p* (P27636), *Hpo* (Q8T0S6), *hMst1* and *hMst2* (Q13043, Q13188) from Universal Protein Knowledgebase (Uniprot, https://www.uniprot.org/).

## Conflicts of Interest

The authors declare no conflicts of interest.

## Supporting information


**Figure S1:** Sequence comparison of the kinase domains between FvM4K1 of woodland strawberry and Ste20 proteins from Arabidopsis, yeast, fruit fly and humans.
**Figure S2:** pbi70286‐sup‐0001‐Figures.pdf. FvM4K1 rescues the growth defects of Arabidopsis mutant *sik1‐4*.
**Figure S3:** pbi70286‐sup‐0001‐Figures.pdf. Identification and observation of vegetative and reproductive organs of M4K1‐RNAi transgenic plants.
**Figure S4:** pbi70286‐sup‐0001‐Figures.pdf. Identification and observation of vegetative organs and achenes (seeds) of M4K1‐OE transgenic plants.
**Figure S5:** pbi70286‐sup‐0001‐Figures.pdf. The FvM4K1‐RNAi knock‐down plants have reduced cell size and number in the leaves and petals while M4K1‐OE have increased in leaves.
**Figure S6:** pbi70286‐sup‐0001‐Figures.pdf. FvM4K1 is autophosphorylated and its kinase activity is required for organ size control.
**Figure S7:** pbi70286‐sup‐0001‐Figures.pdf. FvM4K1 interacts with FvMOB1s in the Hippo pathway.
**Figure S8:** pbi70286‐sup‐0001‐Figures.pdf. Defect of receptacles (fruits) of M4K1‐RNAi.
**Figure S9:** pbi70286‐sup‐0001‐Figures.pdf. Expression profile monitored by RT‐qPCR of *FvM4K1* in different tissues and receptacles at different developmental stages.

**Table S1:** Primers used in this study.
**Table S2:** pbi70286‐sup‐0002‐Tables.docx. Phenotypic analysis of RNAi and OE strawberry plant lines.

## Data Availability

The data that support the findings of this study are available on request from the corresponding author. The data are not publicly available due to privacy or ethical restrictions.
