# Supplementary material for: The Ste20 Homologue FvM4K1 Regulates Organ Size via Hippo Signalling Pathway in Woodland Strawberry ( Fragaria vesca )
Source: Plant Biotechnol J. 2025 Aug 4;23(11):5094–109. doi: 10.1111/pbi.70286 (PMC12576436; doi:10.1111/pbi.70286)
Supplement: Supplementary file 2 — Table S1: Primers used in this study. Table S2: pbi70286‐sup‐0002‐Tables.docx. Phenotypic analysis of RNAi and OE strawberry plant lines. [file PBI-23-5094-s001.docx]

Supplemental information

Table S1 Primers used in this study

| **Name** | **Sequence (5’ to 3’)** | **Purpose** |
| --- | --- | --- |
| attB_M4K1-F | GGGGACAAGTTTGTACAAAAAAGCAGGCTCCACC ATGGCTTTCTTATTCTTCCTC | Full length FvM4K1 CDS in pDONR |
| attB_M4K1-R | GGGGACCACTTTGTACAAGAAAGCTGGGTC CAAACGAAGAATGGTTCTTAATG |  |
| Fvactin-F | GGGCCAGAAAGATGCTTATGTCGG | qPCR |
| Fvactin-R | GGGCAACACGAAGCTCATTGTAGAAG |  |
| qM4K1-F | TCAGATAATGTTCTCGGAGG | qPCR |
| qM4K1-R | ATAGAGTTGGACTTGAATGGA |  |
| qMOB1A-F | ATGAGTCTCTTCGGTCTG | qPCR |
| qMOB1A-R | AGAAATCCACAGTGTTGAC |  |
| qMOB1B-F | GCAGGACCAAAGTATGAG | qPCR |
| qMOB1B-R | ACGGAACATTCGCTTAAAG |  |
| LBb1.3 | ATTTTGCCGATTTCGGAAC | T-DNA left border primer |
| sik1-4_LP | GATCATAT GGATCTGTAT ACAAGGC | *sik1-4* T-DNA line genotyping |
| sik1-4_RP | CAT CCA ATG TGG AGT CCC AAT G |  |
| 35sp-F | CGTAAGGGATGACGCAC | Identification of transgenic Arabidopsis |
| M4K1-R1 | TCTCTCTCACTCGGCCTCATC | Identification of transgenic Arabidopsis |
| M4K1-R2 | CCTTCAATGCTTCCCTGCAAATG | Identification of OE strawberry |
| CmR-R | AGTACTGCGATGAGTGGC | Identification of RNAi strawberry |
| RNAiattB_M4K1-F | GGGGACAAGTTTGTACAAAAAAGCAGGCTTC AAACCCGACCTCTACTCCAC | RNAi fragment in PDONR221 |
| RNAiattB_M4K1-R | GGGGACCACTTTGTACAAGAAAGCTGGGTA GCCGAAGTCCTTGGGAAG |  |
| M4K1_K269E-F | GAGATGGTGGCGATCGAAGTCATTTCATTATCTC | M4K1 phosphorylated mutant |
| M4K1_K269E-R | GAGATAATGAAATGACTTCGATCGCCACCATCTC |  |
| M4K1_T396A-F | GACCATGTCAAAGCGCAATGCATTCATTGGGACTCCACATTG | M4K1 phosphorylated mutant |
| M4K1_T396A-R | CAATGTGGAGTCCCAATGAATGCATTGCGCTTTGACATGGTC |  |
| attB_∆NM4K1-F | GGGGACAAGTTTGTACAAAAAAGCAGGCTCCACCACGAAGTACGAGCTGCTC | Full length FvM4K1 lacking N-terminus |
| attB-M4K1-N-R | GGGGACCACTTTGTACAAGAAAGCTGGGTCATGAATTTGTGCTTCAACATC | Y2H or BiFC |
| attB-M4K1-N1-R | GGGGACCACTTTGTACAAGAAAGCTGGGTCAGTCGGATCTTCTCTGGTG |  |
| attB-M4K1-N2-R | GGGGACCACTTTGTACAAGAAAGCTGGGTCATGAATTTGTGCTTCAACATCTCAG |  |
| attB-M4K1-C-F | GGGGACAAGTTTGTACAAAAAAGCAGGCTCCACCTGAAAAATGCAAATGTGGTCCC |  |
| attB-M4K1-C-R | GGGGACCACTTTGTACAAGAAAGCTGGGTCCTACAAACGAAGAATGGTTCTTAATG |  |
| attB-FvMOB1A-F | GGGGACAAGTTTGTACAAAAAAGCAGGCTCCACCATGAGTCTCTTCGGTCTGG |  |
| attB-FvMOB1A-R | GGGGACCACTTTGTACAAGAAAGCTGGGTCTCAATAAGGAACGATGATAGATTC |  |
| attB-FvMOB1B-F | GGGGACAAGTTTGTACAAAAAAGCAGGCTCCACCATGAGTCTCTTTGGTCTTGGAAG |  |
| attB-FvMOB1B-R | GGGGACCACTTTGTACAAGAAAGCTGGGTCTCACTTTGGCTGTAGAATAGAGTC |  |
| FvMOB1A_T35A-F | GAGAAAGCACATTGATGCAGCACTAGGCAGTGGGAACCTAAG | FvMOB1A phosphorylated mutant |
| FvMOB1A_T35A-R | CTTAGGTTCCCACTGCCTAGTGCTGCATCAATGTGCTTTCTC |  |
| FvMOB1B_T36A-F | CCAACAACATATTGATGCTGCATTGGGCAGTGGGAACCTTAG | FvMOB1B phosphorylated mutant |
| FvMOB1B_T36A-R | CTAAGGTTCCCACTGCCCAATGCAGCATCAATATGTTGTTGG |  |
| E2F-F2 | CTCTTTGTCTGCTCCACTGTAG | qPCR |
| E2F-R2 | CCTCTCAATCCTAACCACTTG |  |
| GRF-F2 | AAGGAACAAAGGAAGGTGTG |  |
| GRF-R2 | AGTCTCCAAAGAAATGGTGG |  |
| ANT-F2 | AGGACTGAATGCTGTGACC |  |
| ANR-R2 | TCGGAGGACTGAAAGAGC |  |
| CYCB2;1-F2 | AGGAAATACTGGAAATGGAGAG |  |
| CYCB2;1-R2 | AGAGAAACTTTGCTGGTTCAC |  |

Table S2 Phenotypic analysis of other lines in RNAi and OE plants

|  | Plant height (cm) | Leaves area  (cm^2^) | Root length  (cm) | Petal  area  (mm^2^) | Receptacle volume (mm^3^) | Achene width  (μm) | Leaf cell area  (μm^2^) | Leaf cell number |
| --- | --- | --- | --- | --- | --- | --- | --- | --- |
| WT | 5.81±0.31 | 8.44±0.16 | 9.955±0.3 | 25.11±2.91 | 151.51±11.02 | 890.566±37.07 | 928.878±160.26 | 8.99E+05±3.15E+04 |
| EV | 5.87±0.6 | 9.37±0.58 | 9±0.13 | 25.93±3.21 | 137.089±9.98 | 864.151±55.72 | 985.01±231.14 | 8.97E+05±6.65E+04 |
| RNAi  (line 4) | 4.63±0.39 | 4.05±0.52 | 6.53±0.57 | 17.99±1.42 | 68.436±9.52 | 588.679±60.44 | 510.34±49.45 | 7.59E+05±1.13E+04 |
| RNAi  (line 8) | 4.53±0.38 | 4.6±0.25 | 5.91±1 | 18.42±0.94 | 59.92±10.69 | 586.792±62.92 | 498.66±25.84 | 7.46E+05±5.97E+04 |
| WT | 5.15±0.27 | 6.53±0.35 | 9.55±0.15 | / | 162.97±5.28 | 905.714±58.27 | 952.34±24.1 | 6.86E+05±2.11E+04 |
| EV | 5.98±0.52 | 6.55±0.22 | 9.89±0.14 | / | 154.55±17.89 | 899.047±31.67 | 990.8±240.92 | 7.05E+05±1.82E+05 |
| OE  (line 6) | 7.43±0.43 | 12.83±1.1 | 12.86±1.74 | / | 192.85±14.24 | 1005.714±56.62 | 1165.07±177.95 | 1.11E+06±7.87E+04 |
